# Is the Consumption of Added Sugar from Common Beverages Associated with the Presence of Attention Deficit Hyperactivity Disorder Symptoms in Thai Medical Students?

**DOI:** 10.3390/nu15204395

**Published:** 2023-10-17

**Authors:** Nalinee Yingchankul, Chompimaksorn Panuspanudechdamrong, Nuthakul Techapipatchai, Tiphakorn Chanmuang, Pintira Netsiri, Nuntaporn Karawekpanyawong, Krittai Tanasombatkul, Phichayut Phinyo

**Affiliations:** 1 Department of Family Medicine, Faculty of Medicine, Chiang Mai University, Chiang Mai 50200, Thailand; nalineey@hotmail.com (N.Y.); chompimaksorn_p@cmu.ac.th (C.P.); nuthakul_techa@cmu.ac.th (N.T.); tiphakorn_c@cmu.ac.th (T.C.); pintira_n@cmu.ac.th (P.N.); krittaikt@gmail.com (K.T.); 2 Department of Psychiatry, Faculty of Medicine, Chiang Mai University, Chiang Mai 50200, Thailand; nuntaporn.karawek@cmu.ac.th; 3 Center for Clinical Epidemiology and Clinical Statistics, Faculty of Medicine, Chiang Mai University, Chiang Mai 50200, Thailand; 4 Musculoskeletal Science and Translational Research (MSTR), Chiang Mai University, Chiang Mai 50200, Thailand

**Keywords:** ADHD, SSBs, added sugar, consumption, medical students, beverage

## Abstract

Attention deficit hyperactivity disorder (ADHD) significantly affects the well-being of medical students in various aspects. Sugar-sweetened beverages (SSBs) pose a potential risk of ADHD. Our study aimed to determine the prevalence of ADHD symptoms and the association between consumption of added sugar in common beverages and ADHD symptoms in Thai medical students. An online cross-sectional survey was conducted among medical students at Chiang Mai University from May 2022 to April 2023. The consumption of added sugar from common beverages in Thailand was assessed using the Thai Adolescence Sugar Sweetened Beverage Intake (THASSI) questionnaire. An Adult ADHD Self-Report Scale (ASRS) score ≥ 3 identified the presence of ADHD symptoms. Multivariable logistic regression was used for the analysis. Of 441 participants, 29.9% had ADHD symptoms. Daily consumption of added sugar from beverages higher than 25 g/day showed an increased risk of ADHD symptoms (adjusted odds ratio (OR) 1.80, 95%CI 1.15 to 2.84, *p* = 0.011). The same trend was observed when using the sex-specific cutoff points (adjusted OR 1.73, 95%CI 1.10 to 2.73, *p* = 0.018). Higher consumption of added sugar from beverages may increase the risk of ADHD symptoms in Thai medical students. This finding supports the implementation of health policies that promote healthy consumption behaviors among medical students.

## 1. Introduction

Attention deficit hyperactivity disorder (ADHD) is a common psychiatric condition characterized by symptoms including inappropriate levels of inattentiveness, hyperactivity, and impulsivity [1,2]. ADHD is often diagnosed in childhood, and it can persist into adulthood [3]. ADHD can also develop in a late-onset form in young adults, even if they did not meet ADHD criteria in their childhood [4]. Even with the increasing prevalence of ADHD over the years, it remains under-recognized and often underdiagnosed [5]. Among medical students, the estimated prevalence of ADHD symptoms ranges from 25 up to 30% [6,7]. ADHD can affect multiple domains of life in medical students, such as academic performance, work performance, and daily life [8,9,10,11]. There are several factors associated with ADHD and its symptoms, including genetics [12], electronic screen time use [13], depression or anxiety [14], family factors [3], environmental factors [1], sleep problems [15], physical activity [13], and dietary patterns [16]. 

Some evidence suggests a possible association between the consumption of sugar-sweetened beverages (SSBs) or caffeine and ADHD symptoms [17]. Several studies have found that children who consume a higher number of SSBs or energy drinks have a greater risk of developing ADHD symptoms compared to those who consume fewer SSBs [18,19,20]. Among medical students, SSBs are also popular nonprescription stimulants (NPSs) used to enhance academic performance or cope with academic stress [21].

Previous research has often focused on examining the consumption of SSBs and ADHD in children. Most of these studies have not identified the dose–response characteristics of added sugar in relation to ADHD symptoms or suggested practical limits for healthy consumption. It is important to note that ADHD can also manifest in adults [3,5]. Notably, there is limited research concerning ADHD among adult populations, including medical students. Thus, the primary aim of this study was to investigate the prevalence of ADHD symptoms among Thai medical students and to establish whether there is a quantifiable association between the consumption of added sugar from common Thai beverages and the presence of ADHD symptoms. Our secondary aim was to explore other potential ADHD-relevant clinical characteristics that could suggest an increased risk of ADHD symptoms. The findings of this study provide information to support appropriate health policies regarding beverage consumption, with the aim of reducing ADHD symptoms and promoting the learning and well-being of medical students.

## 2. Materials and Methods

### 2.1. Study Design and Participants

We conducted an online cross-sectional survey among medical students at Chiang Mai University from May 2022 to April 2023. The survey was developed using the Research Electronic Data Capture (REDCap) platform [22]. We employed convenience and snowball sampling methods to recruit participants. Throughout the study period, the survey was distributed on various social media platforms. The survey was designed to be anonymous and voluntary, ensuring participant confidentiality. The landing page provided details about the survey and its research objectives. Participants gave informed consent by checking the agreement checkbox and completing the survey. The study protocols were approved by the Institutional Review Board of the Faculty of Medicine, Chiang Mai University (FAM-2565-08761).

The target population consisted of undergraduate medical students in their 1st year to 6th year in the academic year 2022. We excluded participants who reported current active underlying medical conditions, those who had been previously diagnosed with mood disorders such as ADHD, major depressive disorder (MDD), anxiety disorder, and bipolar disorders, and participants who did not complete the Thai version of the Adult ADHD Self-Report Scale (ASRS) screener questionnaire [23].

### 2.2. Data Collection

The online survey consisted of three parts. In the first part, participants were asked to provide information on general demographic characteristics, including age, sex, current academic year, weight, height, body mass index, underlying conditions, and current medication. Additionally, they were asked to provide ADHD-relevant characteristics such as family history of ADHD [12], family income [24], parental education [25], sleeping quality and duration [26], and screen time [27]. The second part included the Thai Adolescence Sugar Sweetened Beverage Intake (THASSI) questionnaire version 3 [28], while the third part consisted of the Thai version of the ASRS screener version 1.1 [23]. Both questionnaires had previously been validated in the Thai population [23,28].

### 2.3. Study Determinant

The primary determinant was the total daily amount of added sugar consumed from common beverages, measured in grams (g). To estimate this value, participants were asked to complete the THASSI questionnaire, which required them to provide retrospective information about the beverages they consumed during the previous week. The THASSI questionnaire specifically focuses on 10 common types of beverages, namely sweetened drinks/water, sweetened carbonated soft drinks, energy drinks, coffee, sweetened green tea, sweetened yogurt drinks, sweetened vegetable and fruit juice, sweetened herbal drinks, sweetened soy milk, and sweetened milk [28].

Participants were asked to select the type of packaging (e.g., bottle or can) and size of the beverage they consumed from the provided lists in the THASSI questionnaire. This questionnaire comprehensively covers common beverage products available in the Thai market. They were then required to specify the amount consumed per occasion and the average frequency of consumption per week for each item. The THASSI questionnaire provides the volume of each beverage in milliliters (mL) and the added sugar content per mL for each item. Consequently, the total amount of added sugar from each item can be calculated by multiplying the added sugar content per mL by the total volume consumed within a week. To determine the daily consumption, the weekly consumption amount was divided by 7. Finally, we calculated the total daily consumption of added sugar from all types of beverages by summing up the daily amounts consumed of each type of beverage together.

Since there was no established standard consumption threshold specifically for added sugar from beverages, we divided the total daily consumption for each participant into three equally proportioned tertiles. This categorization enabled us to observe any potential dose–response relationship. Furthermore, we categorized the total daily consumption based on the added sugar limit recommended by the American Heart Association (AHA) [29]. The overall cutoff point proposed was 25 g/day for both sexes. Additionally, we set sex-specific cutoff points at 25 g/day for females and 36 g/day for males [29].

### 2.4. Study Endpoint

The diagnosis of ADHD is typically conducted by a physician using the *Diagnostic and Statistical Manual of Mental Disorders*, Fourth Edition (DSM-IV) [30]. But for the practicality of research, there are several screening tools available for assessing ADHD symptoms in adults. One commonly used tool is ASRS [31], a self-evaluation tool with a few questions developed by the World Health Organization (WHO). The ASRS tool has been validated in multiple languages. The Thai version of ASRS-v1.1 demonstrated good internal consistency, with a Cronbach’s alpha coefficient of 0.92.

The primary endpoint was the presence of ADHD symptoms as determined using the Thai ASRS version 1.1, which serves as a reliable screening tool for adult ADHD [23]. The questionnaire comprises 18 questions that assess both inattentive and hyperactive/impulsive symptoms. In this study, the presence of ADHD symptoms was determined based on the first 6 items of the Thai ASRS [23]. For the first three questions, participants were assigned one point if they reported “sometimes”, “often”, or “very often” as the frequency of occurrence. Similarly, for the remaining questions, participants received one point if they reported “often” or “very often” as the frequency. Participants with a total score of 3 or higher on the first 6 items were considered to have ADHD symptoms. At this cutoff point, the sensitivity and specificity for ADHD diagnosis were 100.0% and 57.5%, respectively [23].

### 2.5. Statistical Analysis

All statistical analyses were performed using Stata version 18 (StataCorp, College Station, TX, USA). Categorical variables were described using frequency and percentage. Numerical variables with normal distribution were described using mean and standard deviation (SD), whereas variables with skewed distribution were described using median and interquartile range (IQR). A non-parametric test for linear trends was used to identify significant trends across total added-sugar consumption tertiles. Fisher’s exact probability test was used to compare the proportions between two independent groups. The Mann–Whitney U test was used to test the differences in non-normally distributed numerical variables between two independent groups. *p*-values less than 0.05 were considered statistically significant, whereas *p*-values between 0.05 and 0.10 were considered borderline significant.

Multivariable logistic regression was employed to investigate the association between the main determinants of interest (total daily consumption of added sugar tertiles, total daily consumption exceeding general AHA-recommended limits, and total daily consumption exceeding sex-specific AHA-recommended limits) and the presence of ADHD symptoms. The model was adjusted for potential confounding variables, including age, sex, BMI, history of ADHD in the family, family income, monthly allowance, maternal and paternal education, sleeping duration, and screen time. Adjusted odds ratios (aORs) and their corresponding 95% confidence intervals (CIs) were reported for each determinant. Additionally, as the total daily consumption represented a combination of ten different beverages, separate analyses were conducted for each one. The consumption amount for each beverage type was categorized into tertiles or median as appropriate. The multivariable logistic models for each beverage type were adjusted with the same confounding variables as the primary model, along with the consumption amounts of the other beverages.

We also conducted an additional analysis to explore the association between other potential factors, including all demographic and ADHD-relevant clinical characteristics, and the presence of ADHD symptoms. Multivariable logistic regression analysis was also employed. Added sugar consumption exceeding the AHA-recommended limits was included in the model as a confounder to be adjusted.

## 3. Results

Of a total number of 1472 medical students invited during the 2022 academic year, 534 (36.3%) responded to the survey. We excluded 19 students who had a history of active underlying medical conditions or had been previously diagnosed with mood disorders including ADHD and 74 students who did not complete the ASRS screener questionnaire. Finally, 441 students were included in the analysis, accounting for 30.0% of all students.

The average age of the students was 20.6, with a male-to-female ratio of 1:1. Almost 60% of the responders were from students in pre-clinical years. Table 1 describes the demographic and ADHD-relevant characteristics of all included participants and also shows the results of the statistical test for linear trends across three groups of students based on their tertiles of daily added sugar consumption. Only a sleep duration less than 7 h showed a significant trend across tertiles. No significant trend of other demographic or ADHD-relevant characteristics was observed across daily added sugar consumption tertiles (Table 1).

Of 441 students, 132 had an ASRS risk score of ≥3. Thus, the prevalence of ADHD symptoms was estimated at 29.9% (95%CI 25.7 to 34.4%). The most prevalent ADHD symptoms identified were trouble wrapping up final details (48.8%), followed by trouble remembering appointments (37.0%) and trouble getting things in order (34.0%). No significant trends of ASRS v1.1 risk score and proportion of students with ADHD symptoms were identified across daily added sugar consumption tertiles (Table 2). However, we observed a significant linear trend of an increasing proportion of participants who had trouble with wrapping up final details across daily consumption tertiles (*p* = 0.048) (Table 2).

From the univariable analysis, students categorized in higher tertiles according to their daily consumption of added sugar from beverages showed a tendency to have ADHD symptoms without statistical significance. Students who reported a consumption of added sugar from beverages higher than 25 g/day regardless of their sex showed a borderline statistically significant association with ADHD symptoms (*p* = 0.061). On the other hand, when the daily consumption was classified based on sex-specific cutoff points (25 g for females and 36 g for males), the association with ADHD symptoms was significant (*p* = 0.046). Table 3 shows the daily consumption of added sugar from all types of beverages separately in students with and without ADHD symptoms. Students with ADHD symptoms reported statistically significant higher consumption of sweetened drinks/water and green tea than students without ADHD symptoms (Table 3).

**Figure 1:**
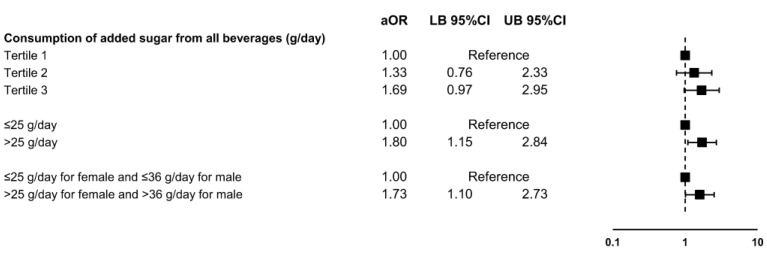
Association between the daily amount of added sugar consumed from all beverages and the presence of ADHD symptoms in Thai medical students. Abbreviations: ADHD, attention deficit hyperactivity disorder; aOR, adjusted odds ratio; CI, confidence interval; LB, lower bound; UB, upper bound.

When adjusted for potential confounding variables, students with a reported daily consumption of added sugar from beverages higher than 25 g/day had a 1.8-times-higher risk of having ADHD symptoms than students who reported a lower consumption (aOR 1.80, 95%CI 1.15 to 2.84, *p* = 0.011). We also identified a significant association between a higher daily consumption of added sugar from beverages and ADHD symptoms when the sex-specific cutoff points were used (aOR 1.73, 95%CI 1.10 to 2.73, *p* = 0.018). Higher tertiles of consumption showed only borderline statistically significant associations with ADHD symptoms (Tertile 2 vs. Tertile 1 aOR 1.33, 95%CI 0.76 to 2.33, *p* = 0.320; Tertile 3 vs. Tertile 1 aOR 1.69, 95%CI 0.97 to 2.95, *p* = 0.063) (Figure 1).

**Figure 2:**
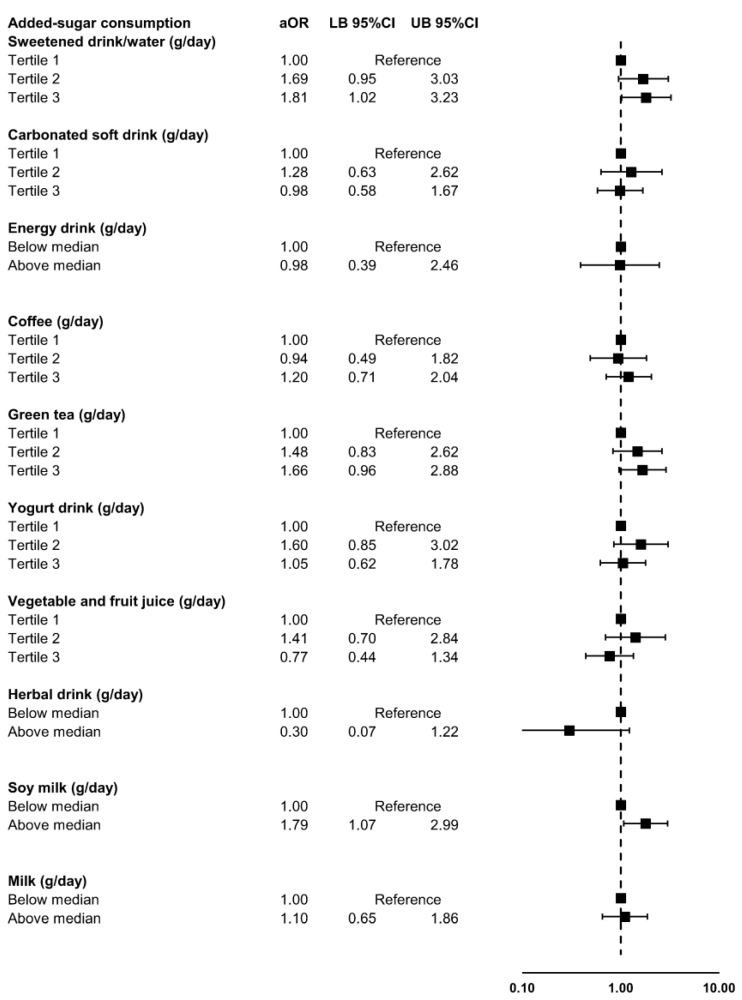
Association between the daily amount of added sugar consumed for each type of beverage and the presence of ADHD symptoms in Thai medical students. Abbreviations: ADHD, attention deficit hyperactivity disorder; aOR, adjusted odds ratio; CI, confidence interval; LB, lower bound; UB, upper bound.

Figure 2 shows the identified association between the daily amount of added sugar consumed for all specific types of beverages and the presence of ADHD symptoms. Higher daily consumption of three types of beverages showed a borderline to statistically significant association with the presence of ADHD symptoms: sweetened drinks/water (Tertile 3 vs. Tertile 1 aOR 1.81, 95%CI 1.02 to 3.23, *p* = 0.043), green tea (Tertile 3 vs. Tertile 1 aOR 1.66, 95%CI 0.96 to 2.88, *p* = 0.071), and soy milk (above median vs. below median aOR 1.79, 95%CI 1.07 to 2.99, *p* = 0.025).

Participants with ADHD symptoms tended to be significantly older, have a higher BMI, be in a higher year of study, and have more daily screen time compared to participants without ADHD symptoms (Table 4). The multivariable analysis revealed that being overweight (aOR 1.92, 95%CI 1.05 to 3.50, *p* = 0.033) and having more than 7 h of daily screen time (aOR 1.97, 95%CI 1.20 to 3.23, *p* = 0.007) significantly increased the risk of ADHD symptoms in our data (Table 5), when adjusting for the amount of added sugar consumed from common beverages.

## 4. Discussion

In this study, up to 30% of medical students in our institution reported significant ADHD symptoms. Higher consumption of added sugar from common beverages was associated with an increased risk of ADHD symptoms. Consuming more than the general daily recommendations of 25 g/day or the sex-specific recommendations (36 g/day for males and 25 g/day for females) may significantly increase the risk of ADHD symptoms. We also identified three types of beverages, sweetened drinks/water, green tea, and soy milk, that have the potential to increase ADHD symptoms. However, these findings still require further confirmation.

The prevalence of ADHD symptoms in our study was found to be 29.9%, closely resembling the reported prevalence of ADHD among medical students in the US in 2019, which was 30.4% [6]. The prevalence of ADHD symptoms among medical students exhibits a wide range, spanning from 3.9% to 37.0% [7,32,33,34]. This variability can be attributed to various factors, such as the diagnostic criteria or diagnostic methods employed, gender differences, and the specific year of the curriculum. When utilizing the ASRS screener to assess ADHD symptom prevalence in medical students, studies have reported rates of 23.7% in Kenya [35] and 37.0% in Brazil [34]. The variability in prevalence rates might be attributed to the distinct cutoff values used to identify ADHD symptoms. In our study, we employed a cutoff based on an ARSR score of 3 or above, which has a sensitivity of 100% and a specificity of 57.5% [23]. The selection of this highly sensitive cutoff was made to ensure that students with ADHD symptoms would not be missed.

It was observed that the consumption of added sugar from common beverages exceeding sex-specific recommendations (i.e., more than 25 g/day for females and more than 36 g/day for males) was associated with an increased risk of ADHD symptoms. This finding aligns with a previous study that reported an association between high consumption of SSBs and ADHD symptoms [17,18]. Interestingly, a previous study reported that added sugar in food alone was not associated with an increased risk of developing ADHD symptoms; only higher consumption of SSBs, or sugary drinks, was [17]. We hypothesized that the amount of sugar added to a general diet might be relatively low and that beverages might be the primary source of consumed sugar.

One potential mechanism through which sugary drinks may impact ADHD symptoms is by disrupting the gut–brain axis pathway. The gut–brain axis serves as a bidirectional communication system between the gastrointestinal tract and the central nervous system. High consumption of sugary drinks has been observed to alter the composition of the gut microbiota due to an unfavorable nutritional environment [36]. This alteration can lead to dysbiosis, gut inflammation, and changes in communication between the gut and the brain via the vagus nerve [37,38]. Such alterations in the gut can result in increased intestinal permeability, contributing to enhanced paracellular transport of lipopolysaccharide (LPS) and other food degradation by-products [37]. LPS is an endotoxin that can translocate across the intestinal epithelium, potentially leading to chronic local or systemic inflammation. In the brain, LPS can bind to Toll-like receptors (TLRs) located on microglial membranes, triggering the activation of the nuclear transcription factor kappa B (NF-kB) and promoting inflammation [36,39]. Microglial activation influences the immune response by producing cytokines and other immune signaling molecules that can interact with the nervous system [37]. Consequently, central inflammation may occur, potentially contributing to ADHD symptoms.

Our study found three types of beverages that may increase the risk of ADHD symptoms. The first involves sweetened drinks/water, which, as the name suggests, is quite straightforward. The predominant component of these beverages is sugar, which may elevate the risk of ADHD symptoms through various described mechanisms, such as disrupting the gut–brain axis pathway. The second type is green tea; our data imply that drinking it may be associated with a higher risk of ADHD symptoms. Our findings differ from those of previous studies that have shown the potential benefits of caffeine and L-theanine, which is found in green tea, in reducing ADHD symptoms [40]. The difference in results may be attributed to the fact that the green tea commonly consumed in Thailand often contains large amounts of sugar, high-fat milk, and added food additives such as artificial food coloring and flavoring agents. Consumption of high amounts of sugar has been shown to impair learning in children and increase aggression and restlessness [41]. Additionally, there is evidence suggesting that children who consume food or beverages containing food additives experience a worsening of ADHD symptoms [42,43,44]. Another beverage identified in our study is soy milk, which was found to notably elevate the risk of ADHD symptoms. Soy milk is one of the non-salicylate foods that children who are sensitive to artificial food colors often show sensitivity too [45]. Such food sensitivities can trigger symptoms of ADHD. These findings indicate that, in addition to added sugar, coloring and flavoring agents could also play a role in the development of ADHD symptoms. Future studies should consider examining the association between these agents and ADHD symptoms. Moreover, since beverages are only a part of what we consume, the association between sugar-sweetened foods and the presence of ADHD symptoms should also be examined.

Besides the amount of added sugar in beverages, two additional factors have been identified as being associated with the presence of ADHD symptoms among Thai medical students: being overweight and having daily screen time exceeding 7 h. The association between overweight/obesity and ADHD has been a subject of conflict for the last two decades [46,47]. It is also possible that the association might be bidirectional, rather than a simple cause-and-effect relationship. Unfortunately, most studies, including ours, were cross-sectional, and thus clear causality could not be implied. Several factors have been suggested to explain this association, including genetics, abnormal eating patterns, impaired executive functions, decreased physical activity and a sedentary lifestyle, altered sleep patterns, and inflammatory mechanisms [46]. Higher daily screen time is another controversial factor for ADHD symptoms [48,49]. It is thought to be interconnected with obesity, as increased screen time leads to a sedentary lifestyle, reduced physical activity, and a positive energy balance [46,48].

This study had several strengths and implications. Firstly, we examined the association between the quantity of added sugar consumption from common beverages in Thai market and ADHD symptoms, specifically among medical students. To the best of our knowledge, this study may be one of the first to examine this important issue in this population and has found that there might be a dose–response relationship between the amount of added sugar in consumed beverages and the presence of ADHD symptoms. Most importantly, using AHA-recommended cutoff values to determine inappropriate amounts of added sugar could provide clearer guidance to promote healthier beverage consumption among medical students. Secondly, data collection for the determinants and outcome was performed using standardized tools validated in the Thai population. However, there are also limitations to consider. Firstly, the proportion of sugar in Thai beverages does not have a standardized measurement, which may have resulted in some inaccuracies when assessing sugar consumption through the questionnaire. Secondly, the study design was cross-sectional, and as a result, causality between the associated factors and ADHD symptoms could not be determined. The design also limits our ability to examine the effect of added-sugar consumption on ADHD symptoms in a longitudinal aspect. Thirdly, due to this study’s design, there might be some recall bias. A prospective study could enhance the accuracy of the data. Fourthly, this study was conducted within the context of the Faculty of Medicine, Chiang Mai University, and the findings may not be generalizable to other communities or contexts. Therefore, future studies should aim to include larger sample sizes and diverse cultural and contextual settings to further investigate this relationship. Finally, since our research domain was focused entirely on medical students, the association between the amount of added sugar from common beverages consumed and the presence of ADHD symptoms identified in this study might not be generalizable to other strata of society. It is important that the existence of this association be examined and confirmed both in the general population and among subgroups within it. While evidence of an association in the general population can inform public health policy, it does not necessarily imply that all underlying subgroups are affected in the same way. Analyzing data from specific subgroups can spotlight particular segments of the population that may be at higher risk than average.

## 5. Conclusions

Increased consumption of added sugar from common beverages may elevate the likelihood of ADHD symptoms among Thai medical students. Our findings highlight the critical need to promote healthier consumption habits among this population specifically. Adopting such measures could potentially mitigate ADHD symptoms among medical students, thereby enhancing their overall well-being and quality of life.

## Figures and Tables

**Table 1:** Demographic and ADHD-relevant characteristics of the included participants.

	Overall	Total Added Sugar Consumption from Common Beverages in g/Day			*p*-Value
		Tertile 1	Tertile 2	Tertile 3	
	(*n* = 441)	(*n* = 147)	(*n* = 147)	(*n* = 147)	
Sex (*n* (%))					
Male	178 (40.4%)	59 (40.1%)	54 (36.7%)	65 (44.2%)	0.476
Female	263 (59.6%)	88 (59.9%)	93 (63.3%)	82 (55.8%)	
Age (years, mean ± SD)	20.6 ± 1.9	20.8 ± 1.9	20.3 ± 1.9	20.5 ± 2.0	0.237
Weight (kg, mean ± SD)	59.3 ± 13.1	60.5 ± 13.9	58.3 ± 11.8	59.0 ± 13.6	0.342
Height (cm, mean ± SD)	165.4 ± 8.2	165.4 ± 8.1	164.4 ± 7.7	166.5 ± 8.5	0.261
BMI (kg/m^2^, mean ± SD)	21.5 ± 3.7	22.0 ± 4.0	21.5 ± 3.4	21.2 ± 3.8	0.072
Year of study (*n* (%))					
1st	103 (23.4%)	26 (17.7%)	40 (27.2%)	37 (25.2%)	0.140
2nd	53 (12.0%)	19 (12.9%)	18 (12.2%)	16 (10.9%)	
3rd	102 (23.1%)	33 (22.4%)	32 (21.8%)	37 (25.2%)	
4th	79 (17.9%)	28 (19.0%)	26 (17.7%)	25 (17.0%)	
5th	38 (8.6%)	15 (10.2%)	14 (9.5%)	9 (6.1%)	
6th	66 (15.0%)	26 (17.7%)	17 (11.6%)	23 (15.6%)	
Clinical year (*n*(%))	183 (41.5%)	69 (46.9%)	57 (38.8%)	57 (38.8%)	0.156
History of ADHD in family (*n* (%))	11 (2.5%)	2 (1.4%)	5 (3.4%)	4 (2.7%)	0.455
Maternal education (*n* (%))					
Below bachelor’s degree	98 (22.3%)	37 (25.3%)	28 (19.0%)	33 (22.4%)	0.592
Bachelor’s degree	251 (57.0%)	83 (56.8%)	82 (55.8%)	86 (58.5%)	
Above bachelor’s degree	91 (20.7%)	26 (17.8%)	37 (25.2%)	28 (19.0%)	
Paternal education (*n* (%))					
Below bachelor’s degree	99 (22.6%)	39 (26.9%)	27 (18.4%)	33 (22.4%)	0.414
Bachelor’s degree	218 (49.7%)	68 (46.9%)	78 (53.1%)	72 (49.0%)	
Above bachelor’s degree	122 (27.8%)	38 (26.2%)	42 (28.6%)	42 (28.6%)	
Monthly allowance (*n* (%))					
Below THB 10,000 per month *	345 (78.2%)	112 (76.2%)	119 (81.0%)	114 (77.6%)	0.778
Above THB 10,000 per month *	96 (21.8%)	35 (23.8%)	28 (19.0%)	33 (22.4%)	
Monthly family’s income (*n* (%))					
Below THB 50,000 per month *	187 (42.4%)	74 (50.3%)	52 (35.4%)	61 (41.5%)	0.125
Above THB 50,000 per month *	254 (57.6%)	73 (49.7%)	95 (64.6%)	86 (58.5%)	
Daily sleep duration (hours, median (IQR))	6 (6–7)	6 (6–7)	7 (6–7)	6 (6–7)	0.088
Daily sleep duration < 7 h (*n* (%))	245 (55.6%)	96 (65.3%)	71 (48.3%)	78 (53.1%)	0.035
Daily screen time (hours, median (IQR))	8 (6–10)	8 (6–10)	8 (6–10)	8 (6–10)	0.723
Daily screen time ≥ 7 h (*n* (%))	309 (70.1%)	106 (72.1%)	100 (68.0%)	103 (70.1)	0.703
Total added-sugar consumption from common beverages (g/day, median (IQR))	24 (13–16)	9 (4–13)	24 (20–28)	46 (36–60)	<0.001

Abbreviations: ADHD, attention deficit hyperactivity disorder; BMI, body mass index; IQR, interquartile range; SD, standard deviation. * Based on the currency exchange rate on June 2023, THB 10,000 was equivalent to approximately USD 288 and THB 50,000 was equivalent to approximately USD 1441.

**Table 2:** Results of the ASRS version 1.1 assessment of the included participants.

	Overall	Total Added-Sugar Consumption from Common Beverages in g/Day			*p*-Value
		Tertile 1	Tertile 2	Tertile 3	
	(*n* = 441)	(*n* = 147)	(*n* = 147)	(*n* = 147)	
ASRS v1.1 risk score (median (IQR))	1 (0–3)	1 (0–3)	1 (0–3)	2 (0–3)	0.156
ADHD symptoms					
Presence	132 (29.9%)	38 (25.9%)	45 (30.6%)	49 (33.3%)	0.162
Absence	309 (70.1%)	109 (74.1%)	102 (69.4%)	98 (66.7%)	
You **sometimes/often/very often** have trouble wrapping up the final details of a project, once the challenging parts have been done	215 (48.8%)	62 (42.2%)	74 (50.3%)	79 (53.7%)	0.048
You **sometimes/often/very often** have difficulty getting things in order when you have to do a task that requires organization	150 (34.0%)	47 (32.0%)	48 (32.7%)	55 (37.4%)	0.325
You **sometimes/often/very often** have problems remembering appointments or obligations	163 (37.0%)	56 (38.1%)	53 (36.1%)	54 (36.7%)	0.809
You **often/very often** have a task that requires a lot of thought, how often do you avoid or delay getting started	92 (20.9%)	24 (16.3%)	38 (25.9%)	30 (20.4%)	0.390
You **often/very often** fidget or squirm with your hands or feet when you have to sit down for a long time	102 (23.1%)	32 (21.8%)	28 (19.0%)	42 (28.6%)	0.167
You **often/very often** feel overly active and compelled to do things, like you were driven by a motor	35 (7.9%)	13 (8.8%)	9 (6.1%)	13 (8.8%)	1.000

**Abbreviations:** ADHD, attention deficit hyperactivity disorder; ASRS v1.1, adult ADHD Self-Report Scale version 1.1; IQR, interquartile range.

**Table 3:** Daily consumption of added sugar from all beverages categorized by the presence of ADHD symptoms.

	With ADHD Symptoms	Without ADHD Symptoms	*p*-Value
	(*n* = 132)	(*n* = 309)	
Daily consumption of added sugar from all beverages (g/day) (*n* (%))			
Tertile 1	38 (28.8%)	109 (35.3%)	0.359
Tertile 2	45 (34.1%)	102 (33.0%)	
Tertile 3	49 (37.1%)	98 (31.7%)	
≤25 g/day	59 (44.7%)	169 (54.7%)	0.061
>25 g/day	73 (55.3%)	140 (45.3%)	
≤recommended cutoff points	68 (51.5%)	191 (61.8%)	0.046
>recommended cutoff points	64 (48.5%)	118 (38.2%)	
Daily consumption of added sugar (g/day) (median (IQR))			
All beverages	26 (13–37)	23 (13–35)	0.162
Only sweetened drinks/water	4 (0–6)	2 (0–5)	0.045
Only carbonated soft drinks	0 (0–4)	0 (0–4)	0.869
Only energy drinks	0 (0–0)	0 (0–0)	0.334
Only coffee	1 (0–8)	0 (0–5)	0.376
Only green tea	2 (0–8)	0 (0–4)	0.030
Only yogurt drinks	3 (0–6)	0 (0–6)	0.668
Only vegetable and fruit juice	0 (0–4)	0 (0–5)	0.513
Only herbal drinks	0 (0–0)	0 (0–0)	0.210
Only soy milk	0 (0–1)	0 (0–0)	0.073
Only milk	0 (0–2)	0 (0–2)	0.999

Abbreviations: ADHD, attention deficit hyperactivity disorder; IQR, interquartile range.

**Table 4:** Demographic and ADHD-relevant characteristics of the included participants categorized by the presence of ADHD symptoms.

	With ADHD Symptoms	Without ADHD Symptoms	*p*-Value
	(*n* = 132)	(*n* = 309)	
Sex (*n* (%))			
Male	54 (40.9%)	124 (40.1%)	0.916
Female	78 (59.1%)	185 (59.9%)	
Age (years, mean ± SD)	20.9 ± 2.1	20.4 ± 1.8	0.015
Age > 20 years (*n*(%))	66 (50.0%)	134 (43.4%)	0.211
BMI (kg/m^2^, mean ± SD)	22.3 ± 4.4	21.2 ± 3.4	0.007
Overweight (*n*(%))	75 (56.8%)	143 (46.3%)	0.048
Year of study (*n* (%))			
1st	26 (19.7%)	77 (24.9%)	0.019
2nd	16 (12.1%)	37 (12.0%)	
3rd	28 (21.2%)	74 (23.9%)	
4th	18 (13.6%)	61 (19.7%)	
5th	12 (9.1%)	26 (8.4%)	
6th	32 (24.2%)	34 (11.0%)	
Clinical year (*n*(%))	62 (47.0%)	121 (39.2%)	0.140
History of ADHD in family (*n* (%))	4 (3.0%)	7 (2.3%)	0.740
Maternal education (*n* (%))			
Below bachelor’s degree	22 (16.7%)	76 (24.7%)	0.158
Bachelor’s degree	79 (59.8%)	172 (55.8%)	
Above bachelor’s degree	31 (23.5%)	60 (19.5%)	
Paternal education (*n* (%))			
Below bachelor’s degree	24 (18.2%)	75 (24.4%)	0.346
Bachelor’s degree	68 (51.5%)	150 (48.9%)	
Above bachelor’s degree	40 (30.3%)	82 (26.7%)	
Monthly allowance (*n* (%))			
Below THB 10,000 per month *	96 (72.7%)	249 (80.6%)	0.078
Above THB 10,000 per month *	36 (27.3%)	60 (19.4%)	
Monthly family’s income (*n* (%))			
Below THB 50,000 per month *	49 (37.1%)	138 (44.7%)	0.171
Above THB 50,000 per month *	83 (62.9%)	171 (55.3%)	
Daily sleep duration (hours, median (IQR))	6 (6–7)	6 (6–7)	0.097
Daily sleep duration < 7 h (*n* (%))	79 (59.8%)	166 (53.7%)	0.251
Daily screen time (hours, median (IQR))	9 (8–11)	8 (6–10)	0.011
Daily screen time ≥ 7 h (*n* (%))	104 (78.8%)	205 (66.3%)	0.009
Daily consumption of added sugar from common beverages > 25 g/day (*n* (%))	73 (55.3%)	140 (45.3%)	0.061

**Abbreviations:** ADHD, attention deficit hyperactivity disorder; IQR, interquartile range; SD, standard deviation; * Based on the currency exchange rate on June 2023, THB 10,000 was equivalent to approximately USD 288 and THB 50,000 was equivalent to approximately USD 1441.

**Table 5:** Exploration of other potential predictors of ADHD symptoms in Thai medical students using multivariable logistic regression analysis adjusted for daily consumption of added sugar from common beverages higher than recommended cutoff point at 25 g/day.

Factors	Adjusted Odds Ratio (95%CI)	*p*-Value
Female	1.04 (0.67 to 1.62)	0.853
Age > 20 years (*n*(%))	0.93 (0.36 to 2.42)	0.879
Overweight (*n*(%))	1.92 (1.05 to 3.50)	0.033
Clinical year (*n*(%))	1.33 (0.51 to 3.47)	0.558
History of ADHD in family (*n* (%))	1.28 (0.35 to 4.65)	0.712
Maternal education (*n* (%))		
Below bachelor’s degree	1.00 (Reference)	
Bachelor’s degree	1.43 (0.71 to 2.85)	0.315
Above bachelor’s degree	1.69 (0.75 to 3.81)	0.208
Paternal education (*n* (%))		
Below bachelor’s degree	1.00 (Reference)	
Bachelor’s degree	1.15 (0.59 to 2.24)	0.675
Above bachelor’s degree	1.16 (0.54 to 2.49)	0.699
Monthly allowance above THB 10,000 per month * (*n* (%))	1.48 (0.88 to 2.49)	0.139
Monthly family’s income above THB 50,000 per month * (*n* (%))	1.02 (0.63 to 1.68)	0.925
Daily sleep duration < 7 h (*n* (%))	1.22 (0.79 to 1.90)	0.367
Daily screen time ≥ 7 h (*n* (%))	1.97 (1.20 to 3.23)	0.007

**Abbreviations:** ADHD, attention deficit hyperactivity disorder; CI, confidence interval. * Based on the currency exchange rate on June 2023, THB 10,000 was equivalent to approximately USD 288 and THB 50,000 was equivalent to approximately USD 1441.

## Data Availability

The data presented in this study are available on request from the correspondent author.
